# Biomimetic Culture Reactor for Whole-Lung Engineering

**DOI:** 10.1089/biores.2016.0006

**Published:** 2016-04-01

**Authors:** Micha Sam Brickman Raredon, Kevin A. Rocco, Ciprian P. Gheorghe, Amogh Sivarapatna, Mahboobe Ghaedi, Jenna L. Balestrini, Thomas L. Raredon, Elizabeth A. Calle, Laura E. Niklason

**Affiliations:** ^1^ Department of Biomedical Engineering, Yale University, New Haven, Connecticut.; ^2^ Department of Anesthesia, Yale University, New Haven, Connecticut.; ^3^ Department of Materials Science and Engineering, Massachusetts Institute of Technology, Cambridge, Massachusetts.; ^4^ Department of Obstetrics, Gynecology, and Reproductive Services, Yale University, New Haven, Connecticut.; ^5^ Department of Pathology, Yale University, New Haven, Connecticut.; ^6^ Raredon Resources, Inc., Northampton, Massachusetts.

**Keywords:** tissue engineering, regeneration, extracellular matrix, bioprocessing

## Abstract

Decellularized organs are now established as promising scaffolds for whole-organ regeneration. For this work to reach therapeutic practice, techniques and apparatus are necessary for doing human-scale clinically applicable organ cultures. We have designed and constructed a bioreactor system capable of accommodating whole human or porcine lungs, and we describe in this study relevant technical details, means of assembly and operation, and validation. The reactor has an artificial diaphragm that mimics the conditions found in the chest cavity *in vivo*, driving hydraulically regulated negative pressure ventilation and custom-built pulsatile perfusion apparatus capable of driving pressure-regulated or volume-regulated vascular flow. Both forms of mechanical actuation can be tuned to match specific physiologic profiles. The organ is sealed in an elastic artificial pleura that mounts to a support architecture. This pleura reduces the fluid volume required for organ culture, maintains the organ's position during mechanical conditioning, and creates a sterile barrier allowing disassembly and maintenance outside of a biosafety cabinet. The combination of fluid suspension, negative-pressure ventilation, and physiologic perfusion allows the described system to provide a biomimetic mechanical environment not found in existing technologies and especially suited to whole-organ regeneration. In this study, we explain the design and operation of this apparatus and present data validating intended functions.

## Introduction

The current standard for treating chronic terminal lung disease is whole-organ transplantation. Although the procedure can give patients many more years of life, transplantation is often delayed due to a shortage of viable organs, and patients who do receive an organ must take immunosuppressive drugs for the rest of their lives. An alternative would be to grow new organs from a patient's own cells, and in 2010, two separate groups showed the potential for *ex vivo* lung regeneration using decellularized whole-organ scaffolds.^1,2^ In these studies, lungs were taken from a rat and perfused with detergent-based solutions that removed cellular material. Such decellularization techniques, when performed with care, leave the extracellular matrix largely intact, preserving the micron-scale histological architecture of the lungs and presenting a fully vascularized whole-organ scaffold.^3–5^ In principle, these scaffolds can then be repopulated with cells derived from the patient, yielding a transplantable artificial organ with little or no immunogenicity.^6^

Specialized bioreactors are essential for this type of whole-organ engineering work, whether it be for lungs or other organs such as livers.^7^ For any organ type, reactors must provide adequate mass transport to all portions of the tissue, control media characteristics such as pH and oxygen, and should provide an organ-specific mechanical environment that approximates the biophysical signals experienced *in vivo*.^8^ In the lung, shear stresses due to perfusion are important mediators of endothelial phenotype,^9^ and cyclic ventilation is known to affect organ development *in utero* and exerts effects on epithelium during regeneration *in vitro*.^10,11^ Any system for the culture of whole lung should therefore allow fine control of both breathing and perfusion, in addition to maintaining sterility and allowing for suitable nutrient and gas exchange.

The predominant commercially available apparatus designed to work with human lungs are *ex vivo* lung perfusion (EVLP) systems, used in the pioneering clinical work of the Lund group^12–16^ and the Toronto group.^17–19^ In EVLP, lungs are taken from a donor, cannulated with flow lines, placed under mechanical perfusion and ventilation in the operating room, and monitored before transplant for relevant outputs such as ΔP_O2_ and vascular resistance. While this process mirrors some aspects of the whole-organ regeneration process, EVLP systems are not well suited for long-term tissue culture. Because EVLP systems are designed for surgical use in an operating room, they do not create a sterile barrier around the organ. During procedures, the lungs are placed on a hard surface and support their own weight, creating a mechanical environment very different from that found in the body; when *in vivo*, the lungs are suspended in fluid and held in place by thin pleural membranes. In addition, these systems use positive-pressure ventilators to breathe the lungs, and there is substantial evidence that positive-pressure ventilation can cause mechanical tissue damage and incite biochemical cascades that negatively alter pulmonary cell behavior and morphology.^20,21^ Although the various designs now used by laboratories for EVLP have some technical variation (see these references^22–27^), no currently published EVLP apparatus is well suited for long-term (e.g., more than 1 day) *ex vivo* whole-lung culture.

Based on the needs described above, we determined a set of functional parameters for a regenerative whole-lung bioreactor. Sterility was of primary importance, and care was taken to build components from materials that could be frequently autoclaved. We designed our system to allow for sterile benchtop maintenance. Since ventilator injury can be somewhat prevented by preserving end-expiratory lung volume through a maintenance of negative pleural pressure,^28^ an overarching goal of this project was to develop an apparatus capable of reliable negative pressure ventilation. Suspending the cultured lung in fluid encased by a “pleural membrane” was deemed preferable, as it more closely mimics the conditions found in the body. Previous studies with small animal models suspended the entire regenerating organ in the culture media. However, at human scale, this technique would have proven prohibitively expensive. We, therefore, aimed to minimize media volume as much as possible and to isolate media to contact with the tissue itself. We also designed the system to be sufficiently compact to be transportable between workstations. Most importantly, the bioreactor had to be capable of mirroring breathing and pulmonary vascular flow profiles corresponding to physiologic profiles, as the utmost goal is to provide a mechanical and chemical environment that delivers appropriate cues to cultured lung cells. The most important functional parameters are delineated in Table 1.

**Table 1.** **Functional Parameters for Regenerative *Ex Vivo* Lung Culture**

Parameter	Required specifications	Commercial EVLP systems
Sterility	Fully sealed, sterile for long culture periods (1–21 days)	Designed for operating room use, open to room air, nonsterile
Ventilation method	Negative pressure	Positive pressure
Ventilation rate	0–15 breaths per minute	Dependent on model and tissue used. Generally, 7–15 breaths per minute, 100 mL·kg^−1^·min^−1^
	0–750 mL per breath	
Ventilation media	Fluid or air	Air
Perfusion method	Pulsatile	Continuous
Perfusion rate	0–90 beats per minute	Dependent on model and tissue used. Maximum flow generally considered 4 L/min
	0–50 mL per beat (up to 4.5 L/min)	
Medium volume outside of tissue	Minimal (<1 L), mechanism to maintain this volume when culturing decellularized (permeable) tissue	0 L (used with intact native tissue)
Disassembly and storage	Allow for benchtop-disassembly and sterile storage of decellularized lung tissue	Not designed for manipulation or storage of lungs outside of operating room
Local tissue environment	Fluid suspension	In contact with air, resting on platform

EVLP, *ex vivo* lung perfusion.

## Materials and Methods

### Bioreactor design

**FIG. 1.**
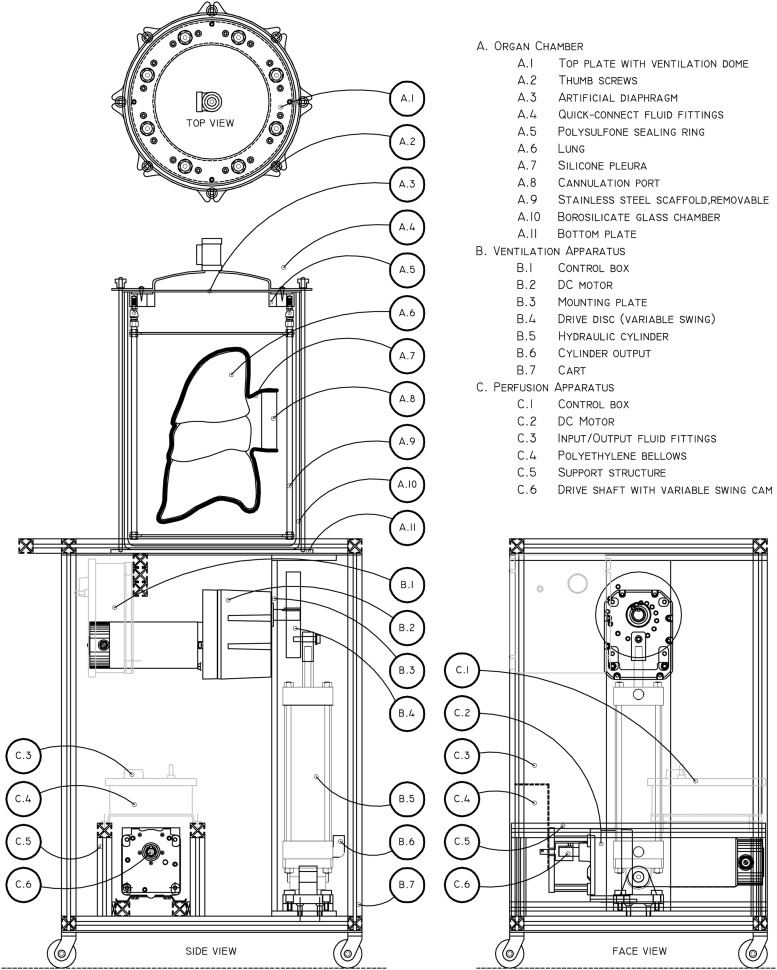
Technical drawings of the bioreactor. The apparatus is composed of three mechanical modules: the organ chamber **(A)**, ventilation apparatus **(B)**, and perfusion apparatus **(C)**. These three modules are linked through ½″ internal diameter tubing (not shown), with flow paths dependent on experimental conditions. The lung is sealed within a silicone pleura (A.7) and any cannulae linked to the cannulation port (A.8, drawn schematically). The construct is then suspended from the removable stainless steel internal architecture (A.9) through loops in the silicone pleura, and flow lines run from the cannulation port to the sealing ring (A.5). Quick-connect fittings (A.6) allow easy management of flow lines to and from the reactor. Oscillation of the hydraulic cylinder (B.5) drives fluid flow in/out of the top plate ventilation dome (A.1), causing flexing of the artificial diaphragm (A.3). Expansion and contraction of the polyethylene bellows pump in the perfusion apparatus (C.4) drives cyclical pulsatile vascular flow. Ventilation can be varied from 0 to 15 bpm at 10–750 mL per cycle. Perfusion can be varied from 0 to 94 bpm at 5–55 mL per cycle. bpm, beats per minute.

The bioreactor consists of a ventilation apparatus, perfusion apparatus, and an organ culture chamber. The breathing and perfusion units are integrated into a wheeled cart that acts as a mobile platform for the organ chamber. Technical drawings of the apparatus are shown in Figure 1.

The organ chamber consists of a 26.5 L glass jar (Fig. 1, A.10), sealed under compression between a top and a bottom plate (A.1 and A.11) clamped with thumbscrews and drop bars (A.2). Handles allow for maneuvering and transport of the filled chamber. The top plate has eight ports for fluid flow, with sterile-disconnect quick-connect fittings (A.4).

Directly beneath the top plate is a silicone membrane (A.3) that generates the seal against the jar/organ chamber. This membrane acts as an artificial diaphragm in the reactor, forming a compliant wall for a variable-volume fluid reservoir in the top plate. The membrane is sealed to the top plate by a custom-machined polysulfone ring (A.5) that isolates the hydraulic fluid behind the diaphragm from the interior of the jar, while allowing fluid flow through the assembly.

Descending from the top plate assembly is a detachable stainless steel architecture (A.9) that helps to position and orient the organ within the organ chamber, providing anchor points for the perfused organ (A.6). This structure doubles as a stand for the upper portion of the organ chamber and the attached organ when disassembled and worked with on a benchtop, allowing manual access to the interior of the organ chamber without physical disruption of the growing construct.

The organ itself is placed within a custom-fabricated silicone pleura (A.7) that is sealed by a 4-inch diameter cannulation port (A.8). The purpose of the silicone pleura is severalfold. First, it creates an isolated media reservoir immediately surrounding the tissue, which minimizes culture medium requirements because it is not necessary to fill the entire glass bioreactor (25 L) with the medium. Second, it holds the organ in a desired position and orientation within the reactor throughout decellularization, seeding, and mechanical conditioning, even when submerged and ventilated with air. And third, it generates a sterile barrier around the organ, allowing the disconnection, storage, and transport of the lung outside of the bioreactor pre- and post-culture. The artificial pleura is highly elastic, made from Shore 10A hardness material with 1,000% elongation at break, which allows it to expand and contract with the organ during perfusion and ventilation. The cannulation port contains junctions for arterial, venous, tracheal, and drainage/pressure adjustment lines. This pleural assembly is one of the key components of the design, which, along with the artificial diaphragm, sets this design significantly apart from other whole-lung perfusion apparatus.

The ventilation apparatus (B) cyclically pumps fluid into and out of the fluid reservoir in the top plate (A.1). Driven by a DC motor coupled to a hydraulic cylinder through a variable-swing eccentric disc (B.2, B.4, and B.5, respectively), the apparatus is capable of pumping fluid volumes between 10 and 1,000 mL in a sinusoidal pattern at rates between 1 and 15 cycles per minute, thereby providing a means to breathe both human- and large animal-sized lungs at physiological rates and volumes. The expansion and contraction of the diaphragm membrane (A.3) drives volume changes in the jar, which subsequently cause the silicone pleura and encased organ to expand or contract, pulling and pushing fluid from an attached reservoir connected to the trachea.

Vascular perfusion is driven by a bellows pump (C.1–C.6) that advances fluid around a vascular loop that includes both a media reservoir and the vascular conduits of the organ. The pump is capable of perfusing 0–55 mL per stroke at rates between 0 and 94 cycles per minute, thereby encompassing most human physiological conditions for lung perfusion. Two one-way silicone poppet/duckbill valves (C.3) direct media flow.

During operation, it is most useful to look at the reactor as a set of five distinct fluid compartments. Supplementary Figure S1 shows a schematic that groups these five compartments, and Supplementary Table S1 details the specific location and purpose of each. The schematic also includes various flow paths, relief valves, check valves, and control consoles necessary for operation.

### Bioreactor construction

#### Organ chamber

**FIG. 2.**
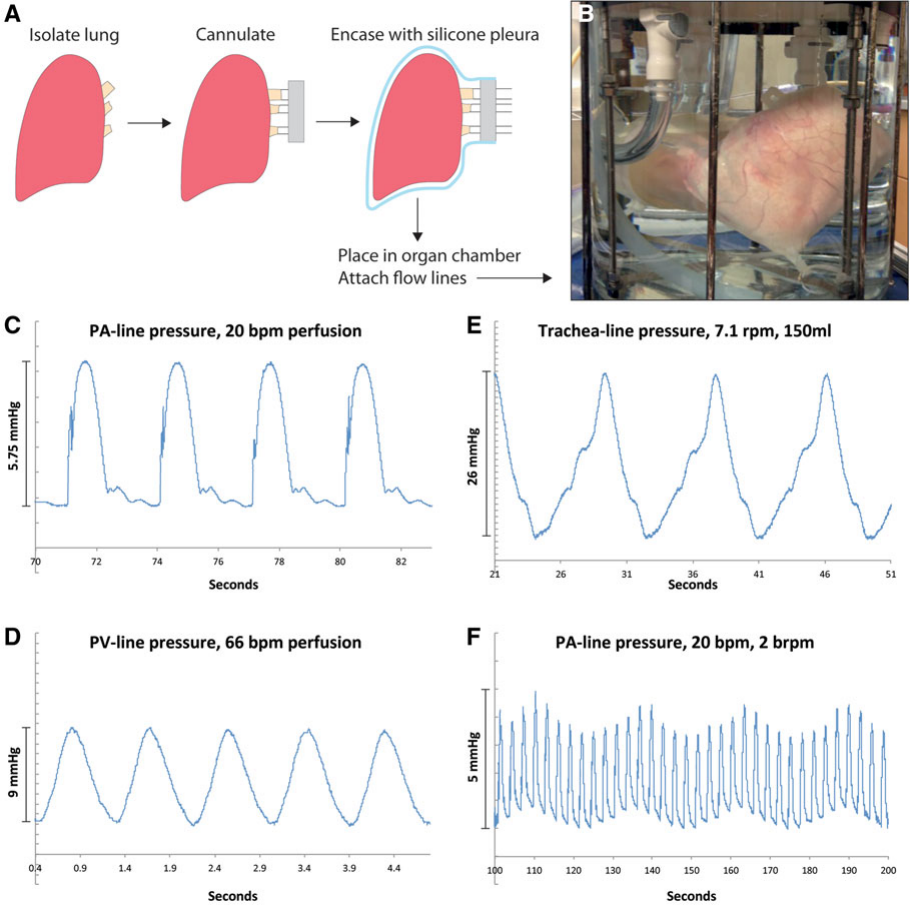
Setup, perfusion, and ventilation. A lung is isolated, cannulated for arterial, venous, and tracheal flow, attached to a cannulation port, and sealed within an appropriately sized silicone pleura **(A)**. Corresponding flow lines are attached, and the assembled construct is placed within the organ chamber and suspended from the internal architecture **(B)**. Arterial perfusion of the construct displays pressure profiles similar to those found *in vivo*, complete with dicrotic notch valve closing **(C)**. Venous outflow displays regular smooth pressure curves **(D)**. Pressure within the trachea flow line oscillates due to negative-pressure ventilation **(E)**. Pressure in the arterial line during simultaneous perfusion and ventilation can be seen in **(F)**, with high frequency oscillations corresponding to 20 bpm perfusion and the lower sinusoidal modulation corresponding to 2 bpm ventilation.

Borosilicate glass (26.5 or 17 L; Simax) jar (A.10) was purchased from Friedrich & Dimmock. Top plate (A.1) and bottom plate (A.11) were laser cut from 1/8″ and 1/4″ aluminum, respectively. Diaphragm dome was hand turned on a lathe out of aluminum sheet to match a machined mandrel designed to have a volume of 0.75 L. Dome and top cap foundation were welded together to create an airtight seal, and ½″ national pipe thread (NPT) fittings attached. All ports were designed to accommodate ½″ NPT to valved quick-connect fittings (A.4; Colder), accommodating ½″ internal diameter tubing. A diaphragm (A.3) was cut from 1/8″ thick platinum-cured silicone to match the top plate port pattern. A sealing ring (A.5) was machined out of high-temperature polysulfone (Radel) to hold this diaphragm to the top plate (A.1), which isolates the space between the dome and the diaphragm while still allowing unimpeded flow through the quick-connect ports; this ring is secured against the top cap by machine screws passing through small holes in the diaphragm. The drop bars and thumbscrews (A.2) were machined from brass and electroplated with nickel. Stainless steel handles were attached to both the top and bottom cap to allow physical transport of the bioreactor unit, and exposed aluminum pieces were powder coated to prevent wear and oxidation (blue in Figs. 2 and 5). The interior anchoring structure (A.9), which would be in contact with fluid, was made either from high-density polyethylene or 316 stainless steel. It consists of four threaded rods that attach through modified quick-disconnect joints to the polysulfone sealing ring and two laser cut crosspieces.

#### Ventilation module

A DC gear motor (B.2, 0–15 rpm, 570 in./lbs torque) was purchased from Bison Gear and Engineering. Three inches diameter hydraulic cylinder (B.5) and associated hardware were purchased from Fabco-Air. A module to mount these components (B.3) was constructed from ½″ steel plate, allowing access to the drive shaft and attachment points for alternate hydraulic cylinders as required. A variable-arm cam (B.4) to join the cylinder to the motor was machined from 1″ aluminum, with offset points drilled at locations corresponding to ventilation volumes at 50 mL intervals from 150 to 750 mL. A control box (B.1) was mounted to allow variable speed control of the motor. The bioreactor cart (B.7) was built around this module from 1″ aluminum extrusion.

#### Perfusion module

A 2½″ diameter bellows metering pump was purchased from Gorman-Rupp and dismantled to salvage the polyethylene bellows (C.4) and silicone poppet/duckbill valves (C.3). A DC gear motor (C.2, 0–94 rpm, 160 in./lbs torque) was purchased from Bison as above. A drive-shaft reducing coupling (C.6) was machined to allow the motor to drive the bellows. A steel mounting bracket was fabricated to bolt the bellows and drive motor together. The assembly was mounted within a portable aluminum-extrusion module designed to lock into the lower part of the ventilation cart (C.5).

#### Silicone pleura and cannulation port

Hollow bronze armatures were handmade in the shape of the desired pleura. Clinical computed tomography scans from de-identified patients were used to determine optimal sizing and spacing of lung lobes, and armatures were created to accommodate a range of patient sizes. These armatures always contained a cylindrical portion that matched the diameter of the desired cannulation port (A.8). Silicone (either Smooth-On Dragon Skin^®^ 10 or Dow Corning Silastic^®^ MDX4-4210) was mixed according to the manufacturer's instructions, degassed, and gently poured over the armatures. The armature was continually rotated during coating to prevent run off. Once the armature was entirely covered, precast silicone attachment loops were pressed into the surface at desired points, and boiling water was pumped through the hollow interior to rapidly cure the polymer. Post curing was completed overnight at room temperature. The cured pleura was then peeled from the armature, cleaned, and trimmed to fit a given cannulation port. The cannulation ports were machined from high-density polyethylene or Teflon and usually contained four barbed fittings on each side, allowing attachment and through-flow of the venous, arterial, tracheal, and pleural lines.

### Lung harvest

All animal experimental work was approved by Yale University's Institutional Animal Care and Use Committee. Yorkshire Swine ranging in weight from 20 to 30 kg were anesthetized using an intramuscular injection of Telazol (4.4 mg/kg), Ketamine (2.2 mg/kg), and Xylazine (2.2 mg/kg). Once anesthetized, animals were administered heparin (300 U/kg) through an intravenous catheter, which was allowed to circulate for 15 min before euthanizing with Euthasol (85 mg/kg). Following euthanasia, an incision was made inferior to the costal margin and the diaphragm was punctured to collapse the lungs. The chest cavity was accessed and the lungs perfused through the pulmonary artery with lactated ringer's solution (Hospira) containing 100 U/mL heparin (Sigma) and 1 μg/mL sodium nitroprusside (Sigma). The lungs, heart, and trachea were excised *en bloc* and placed into 4°C lactated ringer's solution containing 5% penicillin/streptomycin (Gibco), 2% amphotericin B (Sigma), and 1% gentamicin (Gibco) for transport.

### Bioreactor operation

During hydraulically driven negative pressure ventilation, the breathing apparatus cyclically increases and decreases the volume of the breathing compartment fluid found in the upper portion of the organ chamber. This volume oscillation in the upper portion of the organ chamber drives volume oscillations in the lower two reservoirs, the jar reservoir and pleural space, which are both filled with fluid and sealed from the atmosphere. The changes here then cause fluid to be drawn into and pushed out of the lungs from the tracheal reservoir, which is open to atmospheric pressure. The tracheal reservoir takes the form of a large polyethylene media bag. This can be a sterile reservoir for media or air. Separate input and output lines, regulated by one-way valves, minimize trachea-line dead space. This ensures maximum delivery of fresh media or air to the lung during ventilation and allows the medium to be replenished with nutrients and oxygen during fluid breathing. This setup mimics pulmonary negative pressure ventilation-driven *in vivo* by the expansion and relaxation of the rib cage and diaphragm. Vascular perfusion can be either pressure or volume regulated depending on the setup; flow is drawn from a vascular reservoir and is driven into the pulmonary artery by the custom bellows pump.

### Perfusion and breathing demonstration

In a laminar flow hood, lungs were dissected and cannulas were sutured in the pulmonary artery, trachea, and pulmonary veins and connected to the vascular and pulmonary ports. The lung/port assembly was secured inside a sterilized silicon pleura and loaded into the bioreactor chamber. Pulmonary artery and tracheal ports were connected to the bioreactor cap and connected with their respective pumps using 0.5 inch diameter tubing (Masterflex) and quick-connects (Colder). Venous return was collected from the venous cannulae and the pleural space and was returned to the vascular pump through the venous pathway shown in Supplementary Figure S1. TruWave pressure transducers (Edwards) were placed in-line and connected to a PowerLab acquisition system and recorded with the LabChart 5.0 software (ADInstruments). Data were exported into Microsoft Excel (Microsoft) for analysis. Lung cannulation and bioreactor assembly were completed within 2 h of explant.

For short-term experiments, lung vasculature was perfused with either phosphate-buffered saline (PBS) or Lactated Ringer's solution, and the trachea was breathed using either PBS or room air, depending upon the experiment. Vascular flow rates varied from 0 to ∼2 L/min, at pulse rates ranging from 0 to 66 bpm. Breathing rates varied from 0 to 7.1 brpm, with tidal volumes between 0 and 300 mL depending upon the experiment. Demonstration experiments were limited to 3 h in duration per lung.

### 24-Hour bioreactor-mounted lung culture

Left lobes were cannulated, and the bioreactor was assembled in a similar manner as above. Under sterile conditions, the vasculature perfusion lines were connected to the vascular pump and a sterile 20 L reservoir bag, and the perfusion system was loaded with 10 L of Dulbecco's modified Eagle's medium (DMEM) high glucose medium (Gibco) with 10% fetal bovine serum (FBS), 2% penicillin/streptomycin, 0.4% amphotericin B, and 0.2% gentamicin. The airway was filled using the same medium recipe and connected to a separate 20 L reservoir. Media contacting components were either autoclaved or soaked in 95% ethanol for 4 h and left to dry overnight before use. Perfusion was maintained at 120 mL/min (24.5 mL/stroke, 5 bpm), while the breathing diaphragm displacement volume was set at 300 mL and a rate of 2 cycles per minute.

### Histology and immunofluorescence

At the end of the culture period, the bioreactor was disassembled and tissue was sectioned and fixed in 10% natural buffered formalin for 4 h and placed in 70% ethanol overnight, followed by embedding and sectioning into 5 μm slices. Sections were stained with hematoxylin and eosin (H&E) to examine global tissue architecture and cellularity.

For immunofluorescent analysis, slides were deparaffinized using xylene and rehydrated in water. Antigen retrieval was performed in 1 mM EDTA, 10 mM Tris, and 0.05% Tween-20 buffer at pH 9 for 20 min at 75°C and allowed to cool to room temperature for 20 min. After blocking sections with PBS containing 10% FBS and 0.2% Triton X-100 for 45 min, primary antibodies were used against CCSP (07-623, 1:100; Millipore), Pro-SPC (ab40879; Abcam), AQP-5 (AB3559-50UL, 1:500; Millipore), PCNA (ab29, 1:500; Abcam), and PECAM-1 (ab28364, 1:100; Abcam) for 2 h at room temperature. After washing slides with PBS, corresponding secondary antibodies (Alexa Fluor 555) were used at 1:500 dilution for 45 min. Mounting medium containing 4', 6-diamidino-2-phenylindole (DAPI) was applied to slides to visualize nuclei, and coverslips were placed to seal slides. The slides were visualized using a Leica DMI6000 B fluorescence microscope.

### qRT-PCR

Real-time quantitative RT-PCR (qRT-PCR) was used to determine the expression of epithelial and endothelial markers in the native pig lung and the pig lung cultured in bioreactor for 24 h. Portions of lung tissue were taken from each experimental condition (biological replicate *n* = 1) and were used to prepare cDNA for real-time qRT-PCR; PCRs were performed five times in parallel (technical replicate *n* = 5). Total cellular RNA was prepared using the RNeasy Mini Kit following the manufacturer's instructions (QIAGEN). Single stranded cDNA was synthesized using the SuperScript First-Strand Synthesis System according to the manufacturer's protocol (Invitrogen). An equal volume mixture of the products was used as templates for PCR amplification. Reactions were performed in a 25 mL volume with iQ SYBR Green Supermix (Bio-Rad) and 200 nM each of forward and reverse primers using iCycler and iQ software (Bio-Rad). Primers are listed in Supplementary Table S2. Concentrations of all primers were optimized before use. PCR conditions included an initial denaturation step of 4 min at 95°C followed by 40 cycles of PCR consisting of 15 s at 95°C, 30 s at 60°C, and 30 s at 72°C. Threshold cycle (Ct) values from the PCRs for a gene of interest (GOI) were normalized against the Ct values for GAPDH from the same cDNA sample; the results were then averaged to yield the reported mean value and standard error of the mean. Statistical significance was not calculated between conditions as per the guidelines described by Rieu and Powers.^29^ Fold change of GOI transcript levels between sample A and sample B equals 2^−ΔΔCt^, where ΔCt = Ct_(GOI)_ − Ct_(GAPDH)_ and ΔΔCt = ΔCt_(A)_ − ΔCt_(B)_.

### ELISA

A sample of trachea and vasculature effluent (biological replicate *n* = 1) was collected after 24 h of culturing the pig lung in the bioreactor system and used to analyze secreted surfactant protein C values using ELISA Standard Kits from Life Sciences Advanced Technologies according to the manufacturer's instructions. Blank media were run as a control and the resulting average value subtracted from the measurements from collected culture media samples. Technical replicate *n* = 4; error bars shown are standard error across replicates.

## Results

To utilize the bioreactor for lung culture, a freshly isolated and heparinized organ is stripped of excess connective tissue and cannulated with appropriate size tubing and fittings (Fig. 2A). The organ is then placed in the silicone pleura, the pleura is sealed against the cannulation port, and excess air is suctioned from around the tissue. The pleura conforms to the shape of the organ when fluid is suctioned from the space around the lung and maintains an airtight seal with the cannulation port. This assembly, now sealed from the atmosphere, is suspended within the organ chamber and anchored to the interior stainless steel architecture, after which the organ chamber is sealed with the top plate and the glass jar filled entirely with fluid, usually sterile saline. Figure 2B shows the mounted organ at this point, limited within the silicone pleura and mounted to all necessary fittings.

A series of perfusion and ventilation pilot experiments were performed, with pressure transducers placed in the arterial, venous, and tracheal flow lines. All pressure transducers were external to the bioreactor chamber. Selected results of these experiments can be seen in Figure 2C–F, which demonstrate the varying capabilities of both perfusion and breathing in this system. First, a porcine left lung was perfused at 20 bpm, 24.5 mL/stroke, with the airways filled with air and venous drainage into the pleural space. Figure 2C shows the resulting (PA)-line pressure measurements. These curves display physiologic-like patterns, including a visible dicrotic notch from the closure of the poppet valves in the vascular pump. In separate experiments, left and right porcine lungs were mounted in the bioreactor and perfused at 66 bpm, 30 mL/stroke, with cannulated pulmonary veins and fluid-filled airways. Figure 2D shows pressure contours for the resulting venous outflow, which display expected smooth profiles and relatively small pressure changes. Filling the airways with fluid allowed the measurement of tracheal pressures, and Figure 2E shows the pressure profile for the trachea line of a pair of lungs when negative pressure ventilated with fluid at 7.1 cycles/min, 150 mL/cycle. The lower portion of the waveform corresponds to inhalation and upper portion corresponds to exhalation. Continuous simultaneous perfusion and ventilation were also demonstrated, with Figure 2F showing a result from the PA-line cannulated to a left lobe when both perfusion and ventilation are underway, with the narrow peaks corresponding to the vascular perfusion rate and the larger sinusoidal amplitude due to ventilation-induced pressure shifts. Additional relevant pressure contours can be found in Supplementary Figure S2.

**FIG. 3.**
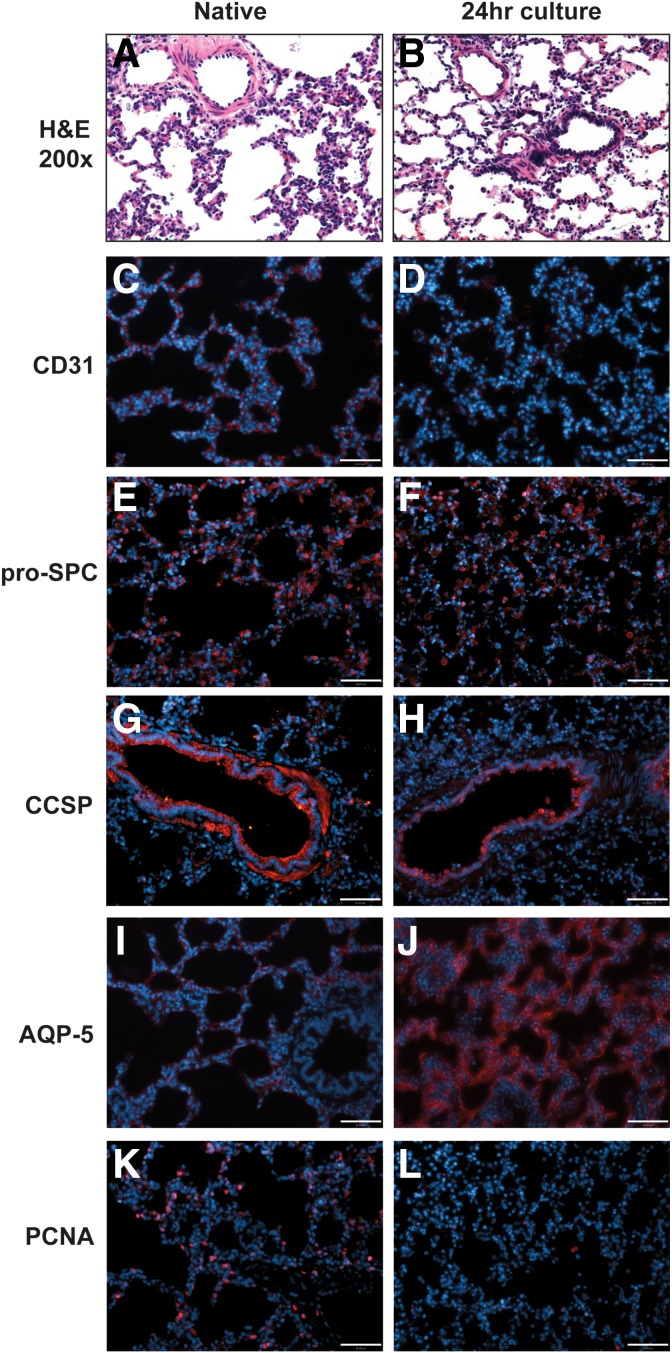
Histology of native lung after 24 h culture. H&E sections showed low levels of tissue damage and maintenance of overall tissue morphology **(A, B)**. *CD31* expression decreased in some alveolar regions **(C, D)**, perhaps due to use of medium that is not optimized for endothelial culture. Epithelial secretion was fairly well maintained in both alveolar (*pro-SPC* staining, **E, F**) and bronchial **(***CCSP* staining, **G, H)** regions. *AQP-5* appeared increased in the cultured lung compared to native **(I, J)**. Cell proliferation decreased during culture, demonstrated by PCNA staining as can be seen in **(K, L)**. H&E, hematoxylin and eosin.

A 24-h culture with continuous perfusion and fluid ventilation was then performed to assess the reactor's ability to support the metabolic demands of fully cellular tissue. A porcine left lung was mounted as described for Figure 1 and was perfused with high-glucose (4,500 mg/L) DMEM at 24.5 mL/stroke, 5 bpm (∼120 mL/min) and ventilated with the same medium mixture at 300 mL/stroke, 2 breaths per min. Sections of the tissue were taken after 24 h of culture at 37°C and were compared to sections from the right lung of the same animal fixed immediately before left lung culture. H&E sectioning (Fig. 3A, B) showed maintenance of tissue morphology, intact alveoli, few dead cells, and normal tissue organization. *CD31* expression was observed more clearly in native as opposed to 24-h cultured lungs (Fig. 3C, D), implying some loss of capillary integrity. This could be due to the used parameters not providing adequate nutrient medium to the overall lung tissue or to insufficient shear stresses in the pulmonary microvasculature. Epithelial survival and secretory markers were well maintained, with *pro-SPC* expression remaining distinct (Fig. 3E–F) and *CCSP* also still clearly visible in the larger airways (Fig. 3G–H), despite the use of fluid ventilation for 24 h. The chosen culture conditions did seem to have a potent effect on aquaporin-5 (*AQP-5*) expression, with the tissue displaying a widespread increase in alveolar expression of *AQP-5* after 24 h of liquid ventilation (Fig. 3I–J). Although the precise cause for the apparent increase in *AQP-5* by immunostaining is unclear, the role of the water channel *AQP-5* in fluid clearance from alveoli may underlie its increase in the setting of liquid ventilation. Cell proliferation decreased following bioreactor culture, as observed through PCNA staining, and may be due to overall low nutrient delivery at the flow rates and volumes used for this experiment (Fig. 3K–L).

**FIG. 4.**
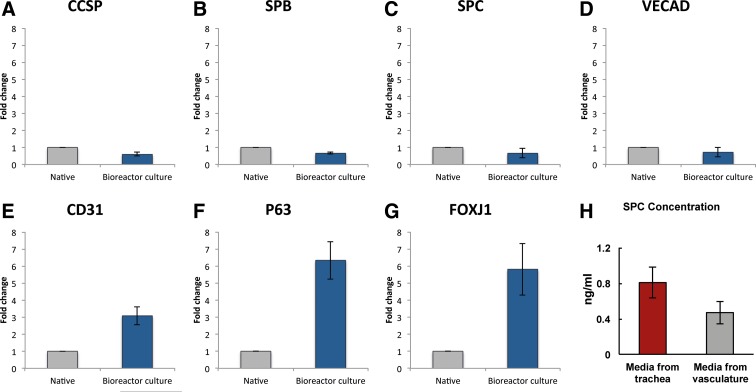
Gene expression by RT-PCR of native lung and after 24 h culture. Corresponding to the histological findings, epithelial secretion was well maintained during culture, as can be seen in measurements of *CCSP, SPC*, and *SPB* expression in **(A–C)**. Endothelial gene expression was also maintained, with VE-cadherin expression and *CD31* expression remaining close to pre-bioreactor values **(D, E)**. A sixfold increase in *p63* expressions was seen **(F)**, suggesting active regulation of proliferation and differentiation of airway precursor cells, which are expected in the setting of lung “injury.” An increase in *FoxJ1*, a marker of ciliation, was observed as well **(G)**. *SPC* concentration was slightly higher in tracheal fluid than in vascular fluid, suggesting a partial mechanical barrier between capillary and alveolar lumens **(H)**. Error bars are standard error of the mean across technical replicates (*n* = 5); however, statistical significance between conditions was not calculated as per the recommendation of Rieu *et al.*, as this data come from multiple samples from a single lung (biological replicate *n* = 1).

Multiple tissue samples were taken from the same experiment and assessed for gene expression using real-time qRT-PCR. Markers of epithelial secretion were well maintained during bioreactor culture, with graphs of normalized *CCSP*, *SPC*, and *SPB* expression shown in Figure 4A–C. Vascular markers showed similar low-fold changes (Fig. 4D, 4E). A sixfold increase in expression of *p63* was seen (Fig. 4F), suggesting that the chosen culture conditions were affecting cell proliferation or development. *FoxJ1* also showed an increase in expression (Fig. 4G), suggesting potential promotion of ciliogenesis. To assess barrier function, a final experiment was performed in which media samples were taken from the tracheal and vascular spaces and surfactant levels compared. ELISA data for this assay can be seen in Figure 4H, showing a higher concentration of secreted components in the airways than in the vasculature, a finding suggesting that there is still an intact mechanical barrier between the physiologic spaces. However, the presence of signal for surfactant protein in the vascular perfusion loops means that there was some breakdown of barrier function compared to native tissue during the 24 h culture period.

**FIG. 5.**
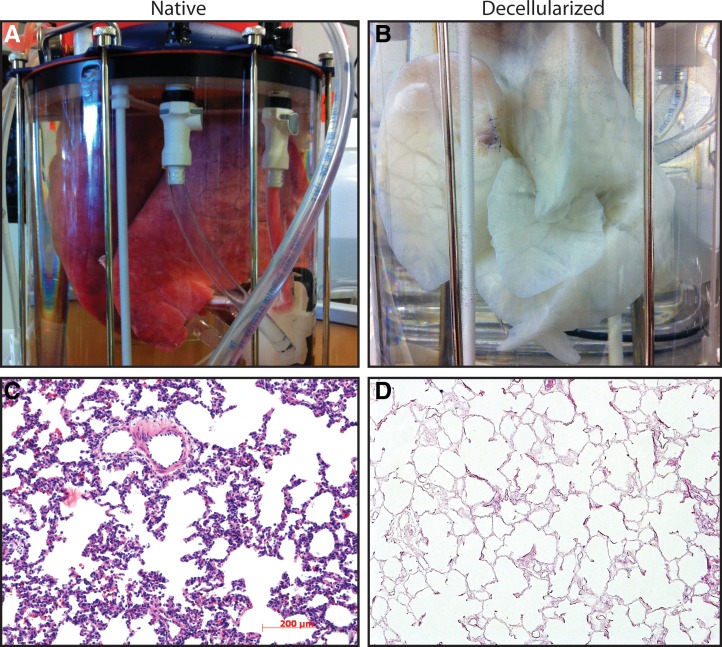
Bioreactor-actuated decellularization. A complete set of porcine lungs was loaded into the reactor immediately following excision **(A)**. The organ was decellularized over a period of 6 h and then rinsed overnight, yielding a robust acellular scaffold **(B)**. Histology from the decellularized organ **(D)** compared to native tissue **(C)** showed successful removal of cells from both the vascular and epithelial spaces and widespread intact matrix architecture.

To assess whether the bioreactor apparatus could be used to decellularize an entire organ, a set of intact porcine lungs were cannulated through the pulmonary artery, veins, and trachea. The lungs were placed within the reactor without a silicone pleura,perfused, and ventilated with previously described decellularizing solutions (Petersen *et al.*^1^). The construct was then rinsed extensively with PBS through both the airways and vasculature and tissue sections taken for histology. The resulting lungs showed consistent decellularization of the entire organ, maintenance of extracellular matrix architecture, and successful removal of all cell types (Fig. 5).

## Discussion

We have developed a system that fills a necessary gap in current technology for whole-organ regeneration. There is currently a critical need in regenerative medicine for advanced functional bioreactors that are scaled for clinical application. We have addressed this need by designing an apparatus specially tailored to human-scale whole-lung decellularization and long-term culture. Particular care has been taken to allow the *ex vivo* creation and maintenance of a macroscopic biomimetic mechanical environment, which is crucial for proper cellular development in the lung.

The artificial diaphragm in the bioreactor is a significant improvement over other *ex vivo* ventilation assemblies, as it allows fine control of negative-pressure ventilation over a wide range of physiologic rates. The perfusion system also sets itself apart from commercially available whole-organ systems as it can achieve vascular flow rates found in the body while displaying pressure contours similar to those generated by the heart. Finally, the incorporation of a flexible pleura around the organ obviates many of the challenges associated with decellularization-based pulmonary regeneration, allowing fluid suspension while helping to maintain sterility and reducing media requirements, which might help this technique to come within financial reach of a broad range of laboratories.

The bioreactor has proven to be a useful tool for decellularization, capable of removing the majority of cellular material with minimal optimization of mechanical parameters.

Histology and RT-PCR suggest that the reactor is able to keep fully cellular tissue alive and functional, although the chosen experimental parameters are clearly not optimized yet to produce an environment identical to that found *in vivo*. It should be noted that culture medium composition plays a significant role in mediating cell morphology and function, and the medium used here as not optimized for epithelial–endothelial coculture. Further studies must be done to determine both optimal media and optimal patterns of mechanical actuation for long-term culture.

We have demonstrated the intended function of the apparatus for a number of use cases, including whole-organ culture and decellularization, as well as breathing and perfusion. We feel confident that the described apparatus will be immediately useful for disciplines involving both whole-organ regeneration and *ex vivo* pulmonary studies. Such specialized technical tools are going to become increasingly important in the coming decades as whole-organ regenerative technology edges toward the clinic. This prototype presents multiple improvements on existing technologies and represents the next step in what we hope to be many generations of iteratively better technologies furthering organ regeneration.

## Supplementary Material

Supplemental data

Supplemental data

Supplemental data

Supplemental data

## Abbreviations Used

bpm: beats per minute
brpm: breaths per minute
DMEM: Dulbecco's modified Eagle's medium
EVLP: *ex vivo* lung perfusion
FBS: fetal bovine serum
GOI: gene of interest
H&E: hematoxylin and eosin
NPT: national pipe thread
PBS: phosphate-buffered saline
qRT-PCR: quantitative reverse transcription polymerase chain reaction
