# Supplementary material for: Biomimetic Culture Reactor for Whole-Lung Engineering
Source: Biores Open Access. 2016 Apr 1;5(1):72–83. doi: 10.1089/biores.2016.0006 (PMC4827315; doi:10.1089/biores.2016.0006)
Supplement: Supplemental data [file Supp_Figure1.pdf]

Supplementary Data

Fluid bodies in the reactor		
Fluid body	*Location*	*Description*
**Ventilation fluid**	Breathing apparatus and top-cap of organ chamber	Breathing apparatus moves fluid to/from the top cap. Flexing of the diaphragm modulates the pressure of the support fluid. Generally tap water.
**Support fluid**	Organ chamber, contacts the diaphragm and surrounds the artificial pleura.	Sealed from the atmosphere and all other fluid bodies. Transfers volume changes from the diaphragm to the organ. Generally non-sterile PBS
**Interpleural fluid**	Between the silicone pleura and the tissue surface. It has a fluid line to the outside of the organ chamber to allow for filling and drainage.	Fluid directly surrounding the organ. As it is not open to the atmosphere, it also acts to transfer diaphragm-driven volume changes to the tissue. Composition dependent on experiment.
**Tracheal fluid**	Inside of airways in the tissue and any attached fluid reservoirs/lines.	Tracheal fluid flow is motivated by diaphragm-driven volume changes of the lung tissue. Upward flexing of the diaphragm draws fluid into the lungs while downward flexing pushes it out. Fluid composition dependent on experiment.
**Vascular fluid**	Inside of the vasculature in the tissue and any attached fluid reservoirs/lines, as well as the interior of the bellows-pump of the perfusion apparatus.	Vascular fluid flow is driven by a in-line metered bellows pump in the perfusion apparatus. Flow can be either pressure- or volume-regulated. Fluid composition dependent on experiment

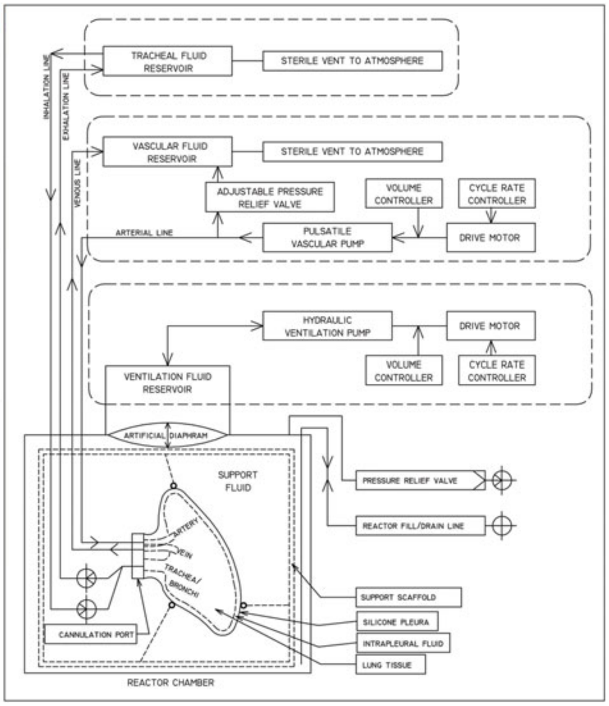

SUPPLEMENTARY FIG. S1. Schematic of fluid flow within the reactor. This diagram details fluid flow paths for the complete bioreactor apparatus. The organ chamber with encased and cannulated lung is represented in the lower left. The lowest dashed area represents the ventilation module, the middle dashed area represents the perfusion module and vascular fluid reservoir, and the upper dashed area represents the tracheal fluid reservoir. Flow lines to/from the organ are clustered on the far left, with flow direction and labels indicated. Selected one-way valves and fill/drain lines have been included as well.
