# Supplementary material for: Biomimetic Culture Reactor for Whole-Lung Engineering
Source: Biores Open Access. 2016 Apr 1;5(1):72–83. doi: 10.1089/biores.2016.0006 (PMC4827315; doi:10.1089/biores.2016.0006)
Supplement: Supplemental data [file Supp_Table1.pdf]

Supplementary Table S1. Fluid Bodies in the Reactor

Fluid body	Location	Description
Ventilation fluid	Breathing apparatus and top cap of organ chamber	Breathing apparatus moves fluid to/from the top cap. Flexing of the diaphragm modulates the pressure of the support fluid. Generally tap water.
Support fluid	Organ chamber, contacts the diaphragm and surrounds the artificial pleura.	Sealed from the atmosphere and all other fluid bodies. Transfers volume changes from the diaphragm to the organ. Generally nonsterile PBS.
Interpleural fluid	Between the silicone pleura and the tissue surface. It has a fluid line to the outside of the organ chamber to allow for filling and drainage.	Fluid directly surrounding the organ. As it is not open to the atmosphere, it also acts to transfer diaphragm-driven volume changes to the tissue. Composition dependent on experiment.
Tracheal fluid	Inside of airways in the tissue and any attached fluid reservoirs/lines.	Tracheal fluid flow is motivated by diaphragm-driven volume changes of the lung tissue. Upward flexing of the diaphragm draws fluid into the lungs, while downward flexing pushes it out. Fluid composition dependent on experiment.
Vascular fluid	Inside of the vasculature in the tissue and any attached fluid reservoirs/lines, as well as the interior of the bellows pump of the perfusion apparatus.	Vascular fluid flow is driven by a in-line metered bellows pump in the perfusion apparatus. Flow can be either pressure or volume regulated. Fluid composition dependent on experiment.

It is easiest to think of the reactor as five interacting fluid compartments. This table outlines these fives spaces, describes where they are within the apparatus, and how they can be modulated to affect one another and provide mechanical conditioning to the organ.

PBS, phosphate-buffered saline.
