# Supplementary material for: Biomimetic Culture Reactor for Whole-Lung Engineering
Source: Biores Open Access. 2016 Apr 1;5(1):72–83. doi: 10.1089/biores.2016.0006 (PMC4827315; doi:10.1089/biores.2016.0006)
Supplement: Supplemental data [file Supp_Table2.pdf]

Supplementary Table S2. Primers Used for qRT-PCR

Gene	Length (bp)	Primer sequences
pSPC	213	Forward: CGCAGAGTATGCCGAGTCTT<br>Reverse: CTTCCTGAGGCCCTGATGTT
pCCSP	76	Forward: CGTCACCTTCACCTTGGTTG<br>Reverse: CTACAAAGCTCGGGCAGAC
pSPB	134	Forward: ACTTTCCGCTGGTCGTTGAT<br>Reverse: CTTGTCCAGCAGAGAGCCT
pFOXJ1	274	Forward: GGATCTTCTGAGGCTGACCC<br>Reverse: GCCGAGGCCTTGAAGCTAAG
pCD31	145	Forward: TCTCATCTGCATCTCGTGGG<br>Reverse: GAAGTGTCAGCAGGACTCCC
pBactin	164	Forward: GTGCGGGACATCAAGGAGAA<br>Reverse: CGTAGAGGTCCTTGCGGATG
PTRP63	109	Forward: GTCAGATCCTGCTACTGCCA<br>Reverse: TGGGTGGGTTCTAGGGTTTC

Single stranded cDNA was synthesized using Invitrogen's SuperScript First-Strand Synthesis System according to the suggested protocol.
