# Supplementary material for: Biomimetic Culture Reactor for Whole-Lung Engineering
Source: Biores Open Access. 2016 Apr 1;5(1):72–83. doi: 10.1089/biores.2016.0006 (PMC4827315; doi:10.1089/biores.2016.0006)
Supplement: Supplemental data [file Supp_Figure2.pdf]

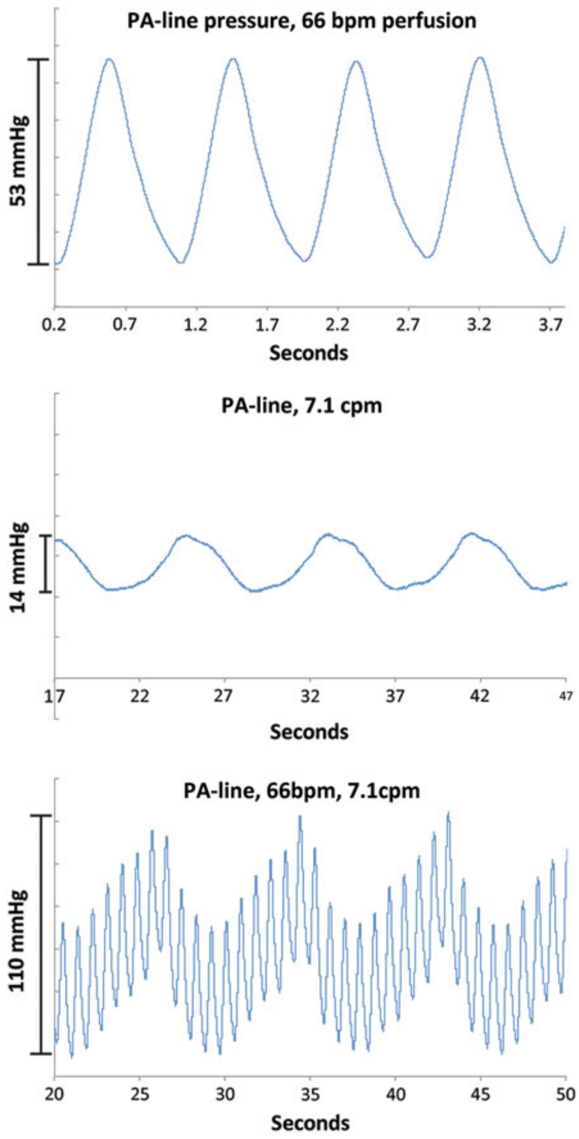

SUPPLEMENTARY FIG. S2. Pressure contours for additional flow/cycle rates. PA-line pressure contours for a complete set of porcine lungs are shown for a higher perfusion rate (top panel, 66 bpm, 30 mL/stroke), physiologic ventilation patterns (middle panel, 7.1 bpm, 150 mL/cycle), and combined simultaneous perfusion and ventilation at the same rates (lower panel). bpm, beats per minute.
